# A Survey of Genomic Studies Supports Association of Circadian Clock Genes with Bipolar Disorder Spectrum Illnesses and Lithium Response

**DOI:** 10.1371/journal.pone.0032091

**Published:** 2012-02-22

**Authors:** Michael J. McCarthy, Caroline M. Nievergelt, John R. Kelsoe, David K. Welsh

**Affiliations:** 1 Veterans Affairs San Diego Healthcare System, San Diego, California, United Sates of America; 2 Department of Psychiatry, University of California San Diego, La Jolla, California, United States of America; 3 Center for Chronobiology, University of California San Diego, La Jolla, California, United States of America; RIKEN Brain Science Institution, Japan

## Abstract

Circadian rhythm abnormalities in bipolar disorder (BD) have led to a search for genetic abnormalities in circadian “clock genes” associated with BD. However, no significant clock gene findings have emerged from genome-wide association studies (GWAS). At least three factors could account for this discrepancy: complex traits are polygenic, the organization of the clock is more complex than previously recognized, and/or genetic risk for BD may be shared across multiple illnesses. To investigate these issues, we considered the clock gene network at three levels: essential “core” clock genes, upstream circadian clock modulators, and downstream clock controlled genes. Using relaxed thresholds for GWAS statistical significance, we determined the rates of clock vs. control genetic associations with BD, and four additional illnesses that share clinical features and/or genetic risk with BD (major depression, schizophrenia, attention deficit/hyperactivity). Then we compared the results to a set of lithium-responsive genes. Associations with BD-spectrum illnesses and lithium-responsiveness were both enriched among core clock genes but not among upstream clock modulators. Associations with BD-spectrum illnesses and lithium-responsiveness were also enriched among pervasively rhythmic clock-controlled genes but not among genes that were less pervasively rhythmic or non-rhythmic. Our analysis reveals previously unrecognized associations between clock genes and BD-spectrum illnesses, partly reconciling previously discordant results from past GWAS and candidate gene studies.

## Introduction

Bipolar disorder (BD) is a serious and life-threatening mental illness that affects 1–2% of the population. Twin and family studies have found that the heritability of BD is up to 0.85, suggesting a strong genetic liability [1]. But despite considerable effort, the precise genetic factors that predispose to BD remain unknown. Based upon clinical observations that patients with BD often exhibit evening chronotype, disturbances in periodic daily activities (e.g. decreased need for sleep, insomnia or hypersomnia, disturbed appetite, and disrupted daily activity patterns), and that mood episodes are affected by light and follow seasonal patterns, a circadian rhythm hypothesis of BD and depression has been developed [2]. This hypothesis has been supported by the development of a mouse model of BD in which a mutation in *Clock*, a core component of the cellular circadian clock, causes lithium-sensitive, mania-like behavioral abnormalities [3]–[4]. Moreover, the mood-stabilizing drug lithium, a mainstay of treatment for BD, also alters clock gene expression [5], and delays circadian rhythms in rodents, monkeys, and humans [6]–[9]. Glycogen synthase kinase 3β (GSK3β) is inhibited by lithium [10], and has been implicated in both lithium's effect on circadian rhythms [11], [12], and its therapeutic action in BD [13].

While it is clear that BD is strongly influenced by genetic risk, and circadian rhythm abnormalities are features of the illness, the genes regulating circadian rhythms (“clock genes”) have yet to be established as an important genetic substrate. On the one hand, candidate clock gene studies of BD have identified a number of variants associated with mood-related phenotypes [14]–[20]. On the other hand, large scale genome wide association studies (GWAS) including three recent meta-analyses [21]–[23], have not identified clock genes as BD associated.

In addition to technical factors (e.g. low density of GWAS markers in candidate genes), at least three fundamental issues could explain the discrepancy in clock gene findings between candidate gene studies and GWAS. First, complex traits are likely to be polygenic. Therefore, individual genetic variants may contribute small effects and/or be weakly associated with the phenotype. For example, a recent GWAS meta-analysis of height identified hundreds of strongly associated polymorphisms that collectively accounted for only a small fraction of the total phenotypic variance [24], suggesting that a large number of weakly associated variants are contributing meaningfully. Of interest, the height-associated genetic loci were non-randomly distributed, showing enrichment in pathways previously implicated in disorders of stature. Therefore BD, like height, may also be caused by a large number of genetic variants, most of which may fail to reach the stringent threshold for genome wide significance [25]. If so, biological pathways may provide helpful structures into which risk-associated variants can be organized. Earlier efforts have already shown some promise in this regard [26], including some implicating clock genes through the convergence of multiple sources of data [27].

A second explanation for the failure to associate clock genes with BD in GWAS may be that the organization of the molecular clock is more complex than previously recognized. While the “core” circadian clock is thought to consist of ∼18 well described genes [28], a recent genome-wide survey identified 343 genes that modulate circadian rhythms when attenuated using siRNA knockdown [29], suggesting that regulation of the circadian clock is governed by a more extended set of genes than previously appreciated. Moreover, downstream of the core clock, thousands of clock controlled genes (CCGs) oscillate rhythmically in at least some tissues [30]. Therefore, circadian rhythms in specific behaviors and physiological processes are likely to be governed not only directly by the core clock, but also indirectly by hundreds of additional genes. It is possible, then, that groups of clock genes or important effector mechanisms of the clock are indeed relevant to BD, and have been collectively implicated by GWAS, but simply not recognized.

A third explanation is that risk for BD conferred by clock genes is distributed across multiple disorders, implying that clock genes may be shared susceptibility genes for multiple illnesses. For example, attention deficit hyperactivity disorder (ADHD) has been associated with evening chronotype, sleep abnormalities and delayed melatonin onset [31]; whereas schizophrenia (SCH) has also been associated with abnormal melatonin rhythms and sleep, as well as variability in daily rhythms [32], [33]. Supporting this hypothesis, candidate studies have broadened the list of psychiatric illnesses with clock gene associations to include ADHD, major depressive disorder (MDD) and SCH [19], [34], [35]. Importantly, clock genes have been reported to regulate brain function more broadly than initially recognized, including the motivation and reward circuits of the midbrain dopamine system [3], [4] and neurogenesis in the hippocampus [36]. If so, clock gene variants may predispose affected individuals to psychiatric illnesses through multiple processes, not just by regulating sleep/wake cycles. The implication of this hypothesis is that the risk for psychiatric illness conferred by clock genes does not fit into existing illness categories, and that illness associations with clock gene variants may be more robust using a broader psychiatric phenotype.

**Figure 1:**
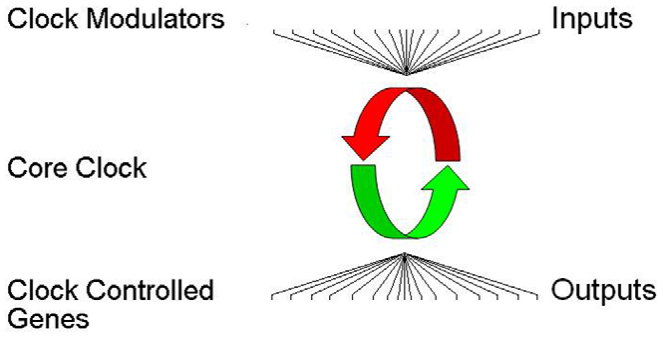
Organization of the extended clock network. The core clock oscillator is a set of ∼18 genes that encode for transcriptional regulators (middle). These proteins are organized in complex feedback loops with positive (green) and negative (red) limbs that generate the ∼24 hr rhythms in gene expression responsible for maintaining circadian rhythms. Upstream clock modulators influence the period and/or amplitude of rhythms by altering protein stability, cellular distribution, or phosphorylation of proteins within the core clock (top). Core clock transcriptional regulators generate expression rhythms in numerous downstream clock controlled genes that are not the “gears of the clock” involved in generating rhythms, but may be important effectors or “hands of the clock” (bottom).

To explore these three possibilities, we conducted a quantitative bioinformatic survey of GWAS results that employed an online repository of genetic data, allowing us to examine multiple GWAS data sets for various psychiatric phenotypes simultaneously. Using this approach, we examined the associations of clock genes with BD and three additional psychiatric conditions for which there is substantial evidence of shared clinical features, genetic overlap and/or enrichment in families of affected BD probands: MDD, SCH and ADHD [24], [37]–[42]. Since circadian rhythm abnormalities are best described in BD, we refer to this group of disorders as the “BD spectrum” of illnesses in order to establish an operational definition, but acknowledge the arbitrariness and limitations of this BD-centered scheme. We also surveyed genes whose expression is reportedly lithium-sensitive in mouse brain [5]. Clock genes were examined at three separate levels: first, 18 “core clock” genes for which an extensive literature supports a critical function in maintaining circadian rhythms; second, an extended set of 342 clock modulator genes that regulate rhythm period and amplitude, but are not involved in the central circadian oscillator; and third, clock outputs, also called clock controlled genes (Figure 1). We found that among the core clock genes there is a statistical over-representation of association with BD-spectrum illnesses in GWAS and lithium sensitive genes. CCGs that were rhythmic in multiple tissues were more likely than weakly rhythmic or non-rhythmic genes to show associations with BD-spectrum illnesses and/or to be lithium-sensitive. In contrast, clock modulator genes were illness associated and lithium responsive only at chance rates.

## Methods

### Psychiatric GWA study repository

GWAS data were identified using the Sullivan Laboratory Evidence Project (SLEP), a searchable online repository of ∼43,000 genetic association entries that integrates genetic information on psychiatric illnesses from disparate sources [43, https://slep.unc.edu/evidence, last accessed January, 2011]. All searches were conducted by gene name. When performed in this way, SLEP search results provide a list of SNPs in the vicinity of a gene of interest, reporting any GWAS evidence of association with selected disorders: BD, MDD, SCH, and ADHD. In some cases, the same SNP may be associated with multiple illnesses. Using this approach, the results of 14 GWAS experiments were queried (Table S1). Results from these searches provided: 1) the study from which the data originate, 2) any associated genetic marker(s), 3) the p-value for the association, and 4) the size of the gene relative to the genome average. Only p-values <10^−3^ are reported by SLEP, and we accepted this value as our threshold for association. While this p-value is not stringent enough to test for genome-wide significance (commonly accepted as p<10^−8^), it may still be pertinent in identifying weakly associated genetic variants that may collectively contribute to illness phenotypes. Candidate gene coverage in GWAS platforms varies considerably. We included in our analysis 100 kb at either end of each gene in order to ensure capture of the majority of *cis* regulatory SNPs [44], [45]. While inclusion of this window could result in more false positive associations, this source of error is the same for test and control genes, negating its contribution to spurious association rates.

### Core clock genes

Eighteen core clock genes were individually selected based on their well-established roles in regulating circadian rhythms in genetic mouse models and/or human sleep disorders. These include: *CRY1/2*, *PER1/2/3*, *NR1D1/2*, *RORA/B/C*, *DEC1/2*, *CSNK1D/E*, *ARNTL1/2*, *CLOCK* and *NPAS2* [28].

### Clock modulators

Clock modulators were defined as the 343 genes affecting circadian period or amplitude in a high throughput siRNA screen [29]. In these 343 cases, siRNA knockdown of the target gene led to a change of >3 standard deviations in period (longer/shorter) or a significant increase in amplitude, as determined by longitudinal (96 hr) measurement of circadian rhythms of gene expression using a *Bmal::dLuc* reporter gene stably expressed in a human osteosarcoma cell line. In most cases, gene names used for SLEP searches were identical to those used previously [29]. In some cases (40 genes, Table S2), an alias used in the initial report was matched manually to the equivalent gene name recognized by SLEP. With this adjustment, all 343 genes were successfully queried in SLEP. Among these 343 clock modulators was one core clock gene *(CRY2).* This gene was therefore excluded, leaving 342 clock modulator genes used in all subsequent analyses.

### Clock controlled genes

Clock controlled genes were selected from a meta-analysis of microarray expression studies identifying 9995 mouse genes that oscillate in at least one of 14 tissues [30]. Among this set, 148 were defined as pervasively rhythmic clock controlled genes (PRCCGs, rhythmic in >6 tissues). This threshold was selected in order to enrich for genes that show widespread rhythmicity, while maintaining adequate sample size. Of the PRCCGs, 10 were previously identified as core clock genes and two others had no human orthologs. After excluding these, the remaining 136 PRCCGs were used in subsequent analyses (Table S2). Weakly rhythmic clock controlled genes (WRCCGs) were defined as one of the 4627 genes that were previously reported to be rhythmic in only one tissue [30]. By comparing the complete list of all rhythmic genes to the list of 8638 genes used for random gene selection, it was also possible to identify 4193 genes lacking evidence for rhythmic expression in any tissue (non-rhythmic genes). For the latter two groups, 450 genes (∼10%) from each set were randomly sampled for analysis in SLEP (Table S2).

### Lithium responsive genes

Genes whose expression in mouse brain is altered by lithium administration have been identified previously using expression microarrays [5]. The full microarray data set was kindly provided in its entirety by the authors. All statistical determinations of lithium responsiveness, including false discovery (FDR) corrections, were conducted by the authors of the original study. SLEP accepts mouse gene entries as the basis for a search, automatically retrieving the corresponding human homolog in the majority of cases.

### Random control genes

Random control gene lists were generated from the 8638 genes present on the Affymetrix Genome Focus microarray with inclusion on the chip determined by the manufacturer. Gene lists were randomly generated with using a publicly available random list generator (http://www.random.org/lists last accessed January 2011). New random gene sets for each comparison were subsequently made for each comparison with replacement such that gene overlap could occur in some cases by chance.

### Statistical analysis

**Figure 2:**
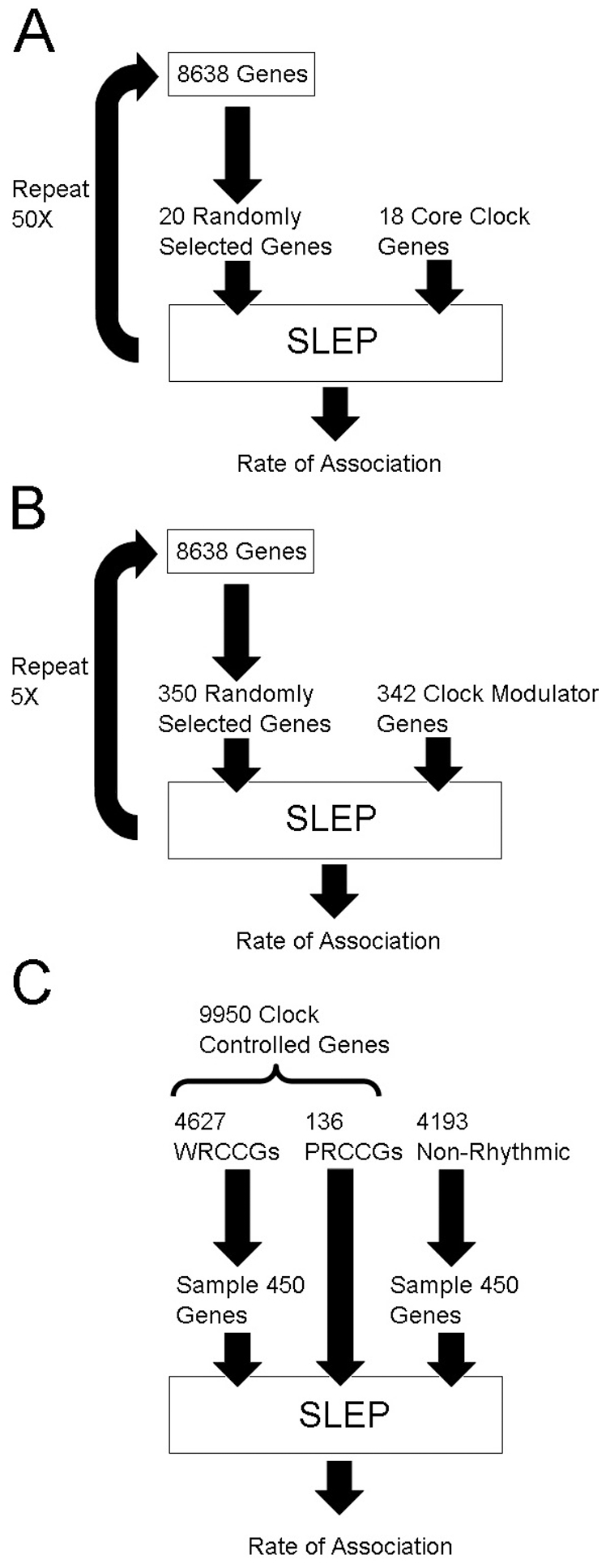
The method for clock gene and random gene analysis is summarized for three test sets. The rate of association in SLEP was determined for sets of randomly selected genes that contained gene numbers similar to A) core clock genes and B) clock modulator genes. C) Pervasively rhythmic (PRCCGs), weakly rhythmic (WRCCGs) and non-rhythmic genes were directly compared. Of 9950 clock controlled genes, 5187 were rhythmic in 2–5 tissues. Genes of intermediate rhythmicity were not examined.

The rate of chance genome-wide association for a gene set in the SLEP database was determined empirically using a series of randomly generated gene sets (Figure 2A, B). For core clock genes, sets of 20 randomly selected genes were submitted to SLEP using the same parameters employed for the core clock genes. Random sets were made slightly longer than test lists to allow for a small number of genes with aliases that did not match the gene name in SLEP. In these cases, genes were excluded from consideration and the association rate was corrected to count only valid searches. For the core clock genes, 50 replications of this process were performed, yielding a total of 948 valid gene searches. For the clock modulator genes, sets of 350 genes were submitted using the same SLEP search parameters. Because the association rate of clock modulators failed to differentiate from the controls, only 5 replications were performed for this gene set. From these base rates, the rate of positive associations for both core clock and modulator genes was compared against the chance rate of the respective control sets. For clock controlled genes, the rates of association between pervasively rhythmic, weakly rhythmic, and non-rhythmic genes were compared directly using methods similar to those described above (Figure 2C). In all cases, a chi-square test was used to test the difference in association rates between clock genes and randomly selected genes, where rate is defined as (associations p<10^−3^/number of genes in list). Odds ratio (OR) values were calculated from the chi square tests. Because only a single hypothesis was considered for each gene set (i.e. the rate of associations in clock genes vs. control genes), no correction for multiple comparisons was required.

### Gene matching

Where indicated, random control genes were matched to clock genes based on relative size. To do so, random genes were run through SLEP without replacement and matched to a clock gene of corresponding size within ±2% of genomic percentile average. The presence or absence of an illness association was then recorded for each control gene. The process was repeated until ten matches per clock gene were made. Subsequent matches beyond those required and unmatched genes were discarded. A power analysis indicated that 10 matches per gene provided >90% power to determine a difference in association rate between core clock genes and controls.

### SNP position and haplotype analysis

Where indicated, detailed positional information for SNPs was obtained using the UCSC genome browser (http://genome.ucsc.edu, March 2006 Assembly), and gene boundaries were defined by the open reading frame posted therein. Haplotype structure was determined using the HapMap II CEU genotype data version 3.2, analyzed using Haploview version 4.2.

## Results

### Core clock gene associations

Among the core clock genes, *ARNTL*, *DEC1*, and *RORB* showed evidence of association with BD (p<10^−3^, Table 1). Interestingly, *DEC1* was associated with BD at two distinct markers in independent studies. When MDD, SCH, and ADHD were considered, an additional six core clock genes (*ARNTL2, NPAS2, NR1D2, PER1, RORA, RORC*) were illness-associated, as well as two additional *DEC1* SNPs associated with MDD and SCH. In total, 9 of the 18 core clock genes (50%) had evidence for genetic association with at least one of the selected psychiatric conditions. Some genes were associated with multiple disorders, so a total of 13 clock gene SNP associations were found. The chance association rate in SLEP for a control set of randomly selected genes was empirically determined to be 3.5 variants per 18 genes (19.5%). The number of chance associations was normally distributed (range: 0–7), and in no case did this number equal or exceed the 9 genes or 13 disorder associations identified using the core clock gene set. Conservatively estimated (i.e. counting an identified gene only once, and not including multiple illness associations), the rate of 50% of core clock genes implicated in BD-related psychiatric illnesses represents a significant enrichment compared to the randomly generated sets [χ^2^(1) = 10.23, OR 4.12, p<0.0015].

**Table 1:** Core clock genes.

Core Clock Gene	Disorder	GWAS p-value (best)	Li FDR p-value
ARNTL	BD	4.39E-04	
ARNTL2	ADHD	6.15E-04	
CLOCK			
CSNK1D			
CSNK1E			
CRY1			**9.80E-05**
CRY2			0.24
DEC1	BD*+MDD+SCH	1.80E-04	0.09
DEC2			
NPAS2	ADHD	6.10E-04	
NR1D1			**0.01**
NR1D2	SCH	8.37E-04	
PER1	SCH	8.71E-05	0.10
PER2			**0.04**
PER3			0.30
RORA	SCH+ADHD	5.62E-05	0.23
RORB	BD	8.99E-04	0.18
RORC	SCH	3.90E-04	

Data shown include 1) Any associations to BD and/or BD-spectrum illnesses as determined by SLEP search (Left) 2) The best GWA p-value for a proximal SNP obtained from SLEP (Middle) 3) A FDR corrected p-value for any gene that was determined to be nominally lithium-responsive (Right). Bold indicates significance after FDR correction.

### Relationship of associated SNPs to core clock genes

The genomic position and haplotype structure of each of the 13 variants identified was then examined in detail to confirm association with a known clock gene. Eight were located in introns of the corresponding clock gene. Two were in probable promoter regions of the clock gene. The remaining three SNPs (those near *PER1*, *NR1D2*, and *RORC*), were located farther from the corresponding clock gene in regions where other genes were likely to be affected (Table 2). In the case of *NR1D2*, the variant rs2001209 was located within a large haplotype block encompassing several genes, and so while rs2001209 lies outside the clock gene, it may tag a haplotype relevant to *NR1D2*. Therefore, the majority of core clock SNPs identified (>10/13) were confirmed to be located within clock genes. For a smaller number of genes (2–3), based on proximity and linkage relationships, we could not rule out a relationship with non-clock genes.

**Table 2:** SNPs with psychiatric illness associations and their location relative to the most proximal clock gene.

Gene	Illness Association	SNP	Position Relative to Gene
PER1	SCZ	rs3027232	Coding region of adjacent gene ALOXE3
NR1D2	SCZ	rs2001209	High density gene area ∼20 kb from NR1D2, intronic in adjacent gene RPL15
RORA	ADHDSCZ	rs922781rs1351546	intronic in RORAintronic in RORA
RORB	BD	rs10869435	intronic in RORB
RORC	SCZ	rs868866	intronic in TDRKH ∼20 kb downstream from RORC
DEC1	MDDBDSCZBD	4 SNPsrs1537720rs11794627rs10982664	Intronic DEC1DEC1 promoter, enriched chromatinIntronic DEC1Intronic DEC1
NPAS2	ADHD	rs11674199	Intronic NPAS2
ARNTL	BD	rs747601	∼20 kb upstream promoter region
ARNTL2	ADHD	rs11049004	Intronic in ARNTL2

The SNPs proximal to PER1 and RORC were determined to lie outside of these genes. The SNP proximal to NR1D2 was indeterminate. The remaining SNPs were determined to be located with the corresponding clock gene.

### Core clock gene size

Gene size may also affect the chance of a spurious genetic association: large genes contain more variants, and thus potentially more false positive associations. Using the SLEP results, we examined the average size of core clock genes relative to the genome average. Core clock genes were larger on average, in the 69.4^th^ percentile of all genes (SD = 23.7). Among core clock genes that were positively associated with a psychiatric disease (compared to those that were not associated), there were more large genes (>85^th^ percentile), and average size tended to be greater (78^th^ vs. 60^th^ percentile), but the overall distribution of associated and non-associated genes was not significantly different. In order to more carefully control for gene size, we repeated our analysis comparing the association rate of the 18 core clock genes to a set of 180 control genes matched to the core clock test set. The chance association rate of the size-matched control genes was 21.6% (39/180 genes), slightly higher than randomly selected genes, but significantly lower than the 50% association rate for core clock genes [χ^2^(1) = 7.15, OR = 3.61, p<0.01], suggesting that gene size alone is not responsible for the observed enrichment.

### GWAS platform and core clock genes

Positive associations for core clock genes were derived from 9 of the 14 eligible studies queried by SLEP, using four distinct platforms (Table S1). This speaks against a platform-specific artifact accounting for our observed results. The SNP density of core clock gene coverage was examined in detail for two of these platforms using annotation on the UCSC genome browser. SNP density among core clock genes was found to be 9.2 kb×SNP^−1^ for Affymetrix 500 K and 5.7 kb×SNP^−1^ for the Illumina HapMap550, compared to genome wide averages of 6.0 kb and 5.5 kb×SNP^−1^ respectively, speaking against an overrepresentation of core clock findings due to SNP selection artifact. Positively associated core clock genes were not more densely covered than genes without association [t(16) = 1.62, p = 0.12].

### Clock modulator gene associations

Among the 342 clock modulator genes, 21 were associated with BD by GWA. Among these, *ATF6* and *CCT5* were also associated with SCH in an independent study. When the psychiatric phenotype was extended to ADHD, MDD, and SCH, an additional 30 clock modulator genes were implicated by GWA, including *FHIT*, which was independently associated with both ADHD and SCH. Combined, a total of 51 of the 342 clock modulator genes (15%) were associated with at least one BD-spectrum illness (Table 3).

**Table 3:** BD-spectrum associated clock modulator genes.

Clock Modulator Gene	Disorder	GWAS p-value (best)	Li FDR p-value
ANKLE1	SCH	3.62E-04	
ATF6	BD+SCH	3.75E-04	
B4GALT2	SCH	1.58E-04	
BAI3	BD	2.91E-04	**0.02**
BLNK	BD	8.27E-04	0.17
BOLA3	MDD	2.27E-04	
BRCA2	BD	2.83E-04	
C16orf82	SCH	5.46E-04	
CCT5	BD+SCH	1.46E-04	
CMTM7	BD	5.49E-05	
EMP2	SCH	6.38E-04	**9.80E-05**
EMR2	MDD	2.56E-05	
EPAS1	SCH	6.58E-04	
FBXL3	SCH	1.53E-04	0.26
FBXO16	SCH	7.72E-04	0.15
FBXW11	BD	7.87E-04	0.08
FHIT	ADHD+SCH	3.71E-04	0.19
FZD10	BD	1.76E-04	
GPR45	BD	7.03E-04	
HAS3	SCH	4.30E-04	
IFNK	BD	1.19E-04	
JAZF1	BD	8.84E-04	
KIAA1797	MDD	5.96E-04	
LHX1	ADHD	1.79E-04	
LSM7	MDD	3.77E-04	
LYG	SCH	1.66E-04	
MAPK8	BD	4.43E-05	0.06
MED30	BD	9.12E-05	
METTL11B	BD	3.66E-04	
MPN2	BD	7.81E-05	
NHP2	SCH	8.38E-04	
OPN5	SCH	6.47E-05	
OXNAD1	ADHD	7.71E-04	
POLR3F	SCH	9.16E-05	
PPM1B	MDD	8.93E-04	
PTK2	BD	4.41E-04	0.25
PWP2	SCH	2.43E-04	
RAB20	BD	5.86E-04	
RCC2	BD	7.05E-04	
RCVRN	SCH	8.62E-04	
RETN	MDD	8.86E-04	
SCARA3	SCH	8.11E-04	
SELO	SCH	8.11E-04	
SH3GL2	BD	4.49E-04	0.31
SPAG4L	MDD	9.56E-04	
SYCP2	MDD	7.45E-05	
tAKR	MDD	4.35E-04	
TBC1D9	BD	3.14E-04	
TUBA1B	BD	2.38E-04	
UNC45B	SCH	1.46E-04	
WDR86	MDD	1.35E-04	

Data shown include: 1) any gene associations with BD or BD-related illnesses as determined by SLEP search (left), 2) the best GWA p-value for a proximal SNP obtained from SLEP (middle), and 3) an FDR-corrected p-value for any gene that was determined to be nominally lithium-responsive (right). Bold indicates significance after FDR correction.

After excluding invalid searches, the chance association rate for a similarly sized set of random genes ranged from 42 to 68 per 350 (mean 57.2±10), or 17.3% (286/1653 tests). Therefore, the association rate of clock modulator genes was not significantly different from the results expected by chance (χ^2^(1) = 1.20, OR 0.83, p = 0.27).

### Clock modulator gene size

Gene size was also examined for the clock modulator genes. Among associated genes, genomic length was in the 57^th^ percentile, whereas among those without GWA associations, genomic length was in the 50^th^ percentile. The size distribution between the two groups was not significantly different (t = 1.649 df = 342, p = 0.10).

### Clock controlled gene associations

PRCCGs, WRCCGs, and non-rhythmic genes were assessed separately for GWA evidence in SLEP. WRCCGs and non-rhythmic genes had a similar number of positive associations in SLEP (75/450 and 79/450 for a rate of 16.6% and 17.5% respectively, p = 0.79, NS). In contrast, PRCCGs had a positive association rate of 25% (34/136, Table 4), reflecting a significant enrichment compared to WRCCGs [χ^2^(1) = 4.79, OR = 1.76, p<0.03], and a trend towards enrichment when compared to non-rhythmic genes [χ^2^(1) = 3.72, OR 1.57, p = 0.05]. Since non-rhythmic genes and WRCCGs did not differ, they were pooled for comparison against PRCCGs, and the enrichment remained significant [χ^2^(1) = 5.62, OR 1.67, p<0.02]. Of 34 genes, 11 were BD associations and the remaining were distributed among ADHD, MDD, and SCH. We also examined whether rhythmic expression in the brain favored illness association among the 136 PRCCGs. PRCCGs that were rhythmically expressed in the SCN, frontal cortex, or whole brain [30] were no more likely to be illness-associated than those that were not rhythmic in the brain (SLEP association rates 24.7% and 25.7% respectively).

**Table 4:** Pervasively rhythmic clock-controlled genes associated with BD-spectrum illnesses.

Clock ControlledGene	Disorder	GWAS p-value (best)	Li FDR p-value
ACSL1	ADHD	6.91E-04	
ANP32A	MDD	6.51E-04	0.25
BTF3L4	SCH	2.46E-04	
CAPRIN1	BD	5.34E-05	
CEBPB	MDD	3.59E-04	
COL4A1	SCH	5.99E-04	0.24
COL4A2	SCH	5.99E-04	
COL6A1	ADHD	5.38E-04	
DUSP1	BD	5.36E-04	0.18
ELOVL5	SCH	6.44E-04	0.21
FOXP1	BD+MDD+SCH	3.43E-05	0.20
GLUL	MDD	9.81E-04	**4.79E-03**
H1F0	ADHD	1.85E-04	0.25
H3F3B	SCH	9.07E-05	**0.02**
HERPUD1	MDD	4.11E-04	**0.02**
HIST1H1C	SCH	3.71E-04	**2.90E-03**
HIST1H2BC	SCH	3.49E-04	0.21
HMGB3	BD	4.05E-04	
MAPRE1	MDD+SCH	1.12E-04	**0.04**
MCL1	ADHD	8.23E-04	0.09
NAMPT	BD+MDD	6.46E-04	
NEDD4L	ADHD	9.34E-05	0.17
PDIA6	ADHD	7.63E-04	
PFKFB3	BD+MDD	7.67E-05	0.15
PSMD11	MDD+SCH	4.28E-04	**0.02**
SASH1	MDD	8.16E-04	0.08
SFRS5	BD	9.66E-04	**0.03**
SFRS6	ADHD+SCH	1.20E-04	
SLC4A4	BD	2.13E-05	0.07
TIMP3	BD	7.16E-05	**0.02**
TUBA1B	BD	2.38E-04	
UBC	MDD	1.34E-04	
ZNF706	BD	3.72E-05	

Data shown include 1) any gene associations with BD or BD-related illnesses as determined by SLEP search (left), 2) the best GWA p-value for a proximal SNP obtained from SLEP (middle), 3) an FDR-corrected p-value for any gene that was determined to be nominally lithium-responsive (right). Bold indicates significance after FDR correction.

### Overlap between clock genes, illness-associated genes, and lithium-responsive genes

Lithium affects circadian rhythms as well as the course of BD. By determining the overlap between clock genes, genes associated with BD-related illnesses, and lithium-responsive genes, a small number of clock genes relevant to both BD and lithium response may be identifiable. Therefore, clock genes (core, modulators, and CCGs) and illness-associated genes were cross-referenced against a set of genes whose expression in the mouse brain is altered by lithium exposure [5] in order to find genes present in all three sets. It was expected that clock genes would be enriched in the overlapping sets relative to randomly selected genes.

**Figure 3:**
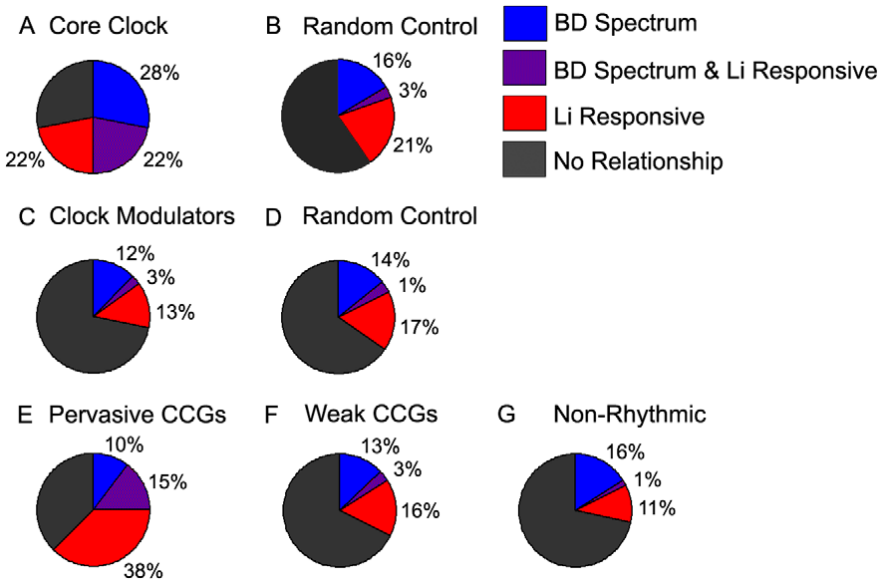
Overlap among clock genes, genes associated with BD spectrum illnesses by GWAS, and lithium-responsive genes. For each set of genes, the % associated with BD spectrum illnesses by GWAS (blue), the % found to be lithium-responsive (red), and the overlap between these (purple) is shown for 18 core clock genes (A), 342 clock modulator genes (C) and 136 pervasively rhythmic clock controlled genes (CCGs) (E) compared to their respective randomly selected controls (B and D), CCGs that are less pervasively rhythmic (F), and genes with no evidence for rhythmicity (G). Gray indicates % of genes with no relationship to either BD or lithium response.

Random control data sets revealed a chance three-way overlap rate of 58/1653 (3.5%). Eight of the 18 core clock genes (44%) were reported as nominally lithium-responsive previously [5], and in our three-way overlap analysis, four of the 18 (22%) core clock genes (*DEC1*, *PER1*, *RORA*, *RORB*) were both nominally lithium-responsive and associated with psychiatric illness by a GWA study in SLEP (Figure 3A, Table 1). However, none remained after employing the FDR correction for determining lithium responsivity [5]. Among the 342 clock modulator genes, 57 (16%, Table S3) were found to be nominally lithium-responsive, but only ten of these (2.9%; *BAI3*, *BLNK*, *EMP2*, *FBXL3*, *FBXO16*, *FBXW11*, *FHIT*, *MAPK8*, *PTK2*, and *SH3GL2*) were both lithium-responsive and associated with psychiatric illness (Figure 3B, Table 2). Of these genes, *BAI3* and *EMP2* were previously reported [5] to be lithium-responsive after FDR correction, with *MAPK8* narrowly missing the significance threshold with an FDR-corrected p<0.06. These data suggest that the core clock is significantly enriched in genes that are both illness-associated and lithium-responsive (22% vs. 3.5%, χ^2^(1) = 17.45 p<0.0001, OR 7.86), whereas clock modulators are not (p = 0.87). Among PRCCGs, 71/136 (52.2%) were nominally lithium-responsive, compared to only 89/450 (19.7%) and 54/450 (12%) for the randomly selected WRCCGs and non-rhythmic gene sets, respectively (Table S3), a significant enrichment among PRCCGs [χ^2^(2) = 103.4, p<0.0001]. Examining the three way overlap between clock controlled genes, illness association, and lithium-responsiveness, PRCCGs had a three way overlap rate of 20/136 (14.7%) compared to only 15/450 (3.3%) and 9/450 (1.9%) respectively for WRCCGs and non-rhythmic genes, a significant difference between PRCCGs and the two other groups [Figure 3C, χ^2^(1) = 24.1 OR 5.0 and χ^2^(1) = 35.84 OR = 8.5, p<0.0001 in both cases). A summary of overlap results after FDR correction is shown in Table 5.

**Table 5:** Summary of the extended clock gene set included in the overlap between BD-spectrum and lithium induction after FDR correction.

Illness	Core Clock	Clock Modulators	Clock Controlled
ADHD	None	None	None
BD	None	BAI3	SFRS5TIMP3
MDD	None	None	GLULHERPUD1MAPRE1PSMD11
SCH	None	EMP2	H3F3BMAPRE1PSMD11

## Discussion

Our findings support the hypothesis that variation within clock genes contributes to BD and related illnesses like ADHD, MDD, and SCH that share genetic and/or clinical features with BD. While none of the individual clock genes examined has been strongly implicated by GWAS, collectively they are associated with BD spectrum illnesses at a rate higher than would be expected by chance, i.e. there are more illness-associated genes among the 18 core clock genes than among similarly sized control sets of randomly selected genes. This enrichment is limited to the core clock genes and their most pervasively rhythmic biological outputs, the PRCCGs, and does not extend to either the modulator genes that have been shown to alter clock period and amplitude [29], or to less pervasively rhythmic genes (WRCCGs). Taking lithium-responsiveness into account, we find a similar pattern of results: genes that are both illness-associated and lithium-responsive are significantly enriched among core clock genes and among PRCCGs, but not among clock modulators or WRCCGs. Collectively, our findings support the hypothesis that the modest associations between BD-spectrum illnesses and single clock genes found in GWAS represent real effects that are too weak to achieve genome-wide significance, and that the clock gene network represents an important point of convergence for BD and the mechanism of action for lithium. Like another recent finding that implicated the clock genes ARNTL and RORB in BD [27], our results demonstrate the value of strategies that integrate disparate sources of biological and genetic data.

The technique used in our study has limitations. The studies in SLEP do not include GWAS data published after 2009 (Table S1), and therefore do not utilize the most recent and presumably, most advanced GWAS results for the disorders of interest. Among these is a GWAS of anti-depressant response in MDD that identified *RORA* among the most strongly associated alleles [46]. Many of these newer GWAS have utilized subjects of non-European ancestry, whereas studies included in SLEP utilized primarily Caucasians. Therefore our searches while extensive, were not comprehensive, and may have failed to detect some associations of interest. Moreover, the results may not generalize to non-Caucasians. The original GWA studies also differ in methodology. However, in all but one study, the number of SNPs tested was similar (435–600 thousand, Table S1), and the sample sizes of studies yielding clock associations and those that did not were not significantly different.

Similar caveats apply to the gene expression data. A number of microarray expression studies have been conducted in rodent brains, but agreement among their results is rare [13], [47]–[49]. In each case, there are differences in lithium administration, the brain regions examined and the microarray platform employed that may explain the divergence of findings. In examining the genes affected by lithium in one of these additional studies, an examination of the rat frontal cortex [49], no core clock genes were represented; only *BMP4*, *DDX3X*, *IKBKB* and *WNT2* overlapped with our list of clock modulators, and only *BHLHB2*, *GJA1*, and *PFKFB3* overlapped with our list of PRCCGs. Of these, only *PFKFB3* was associated with a psychiatric illness in our SLEP search. Therefore, the differences in gene expression implicated in BD/lithium response may vary by experimental condition, making the catalogue of BD/lithium-associated clock genes subject to revision as data emerge.

A further limitation is that because our search strategy required manual data input, the number of searches conducted to establish empirical p-values was relatively small compared to a fully automated approach. Therefore with more repetitions, more precise estimates of chance association could be established. Finally, the haplotype structure of the clock genes could bias our results if they deviated systematically from the rest of the genome. Indeed, certain classes of genes are predisposed towards or against genomic rearrangement, and might be expect to harbor atypical haplotype structures [50], [51]. However, the clock genes have not been previously implicated in this regard [50], [51], and in our own analysis, clock genes show a wide range of haplotype structures, with estimates of mean linkage disequilibrium across the gene (D′) that range from 0.50 to 0.99, and are inversely correlated (r = −0.46) to gene size, typical of haplotype relationships reported elsewhere [50]. Thus, there does not appear to be a systematic bias in clock gene propensity for re-arrangement that could adversely affect our results.

Our findings argue against the hypothesis that GWAS have overlooked strong (i.e. genome-wide significant) associations between BD and previously unrecognized clock genes. However, we did find support for the hypothesis that pathological clock gene variation is distributed across diagnostic categories. Our findings suggest that within the BD-spectrum, clock gene variation is not enriched in any particular disorder, although methodological differences between studies and differences in the number of studies of each illness make these comparisons tentative. In this context, it remains an important issue for future research to further define the rhythm abnormalities in BD-spectrum disorders, more carefully identifying both the unique phenotypes and areas of overlap shared across the illness, then connecting these to specific genetic and neurobiological substrates.

Based on our findings, we conclude that the major influence of the clock on BD spectrum illnesses originates within the core clock and its downstream effector systems, rather than in upstream clock modulators. This suggests that previously noted associations between circadian rhythms and mood disorders not likely to be explained by a common process upstream of both the circadian clock and mood regulatory mechanisms, but rather argues for a more fundamental connection between the clock and mood. The apparent lack of brain specific rhythms among illness-associated PRCCGs is somewhat surprising and points to the possibility of indirect roles in illness progression, perhaps involving immune, endocrine, and/or developmental processes that are not primarily regulated in the brain. Alternatively, this lack of brain specificity would also be expected if the clock's influence on mood involves ubiquitous intracellular signaling pathways found in non-neuronal cells as well as in neurons.

The criteria employed for PRCCGs selection precludes the detailed examination of several CCGs with expression patterns restricted to anatomically discrete regions. Among these are many genes of particular interest to the BD-spectrum, including those involved in monoamine and melatonin synthesis such as *TPH1* and *AANAT*, both of which are expressed rhythmically in a tissue specific manner [30]. Of note, analysis of *TPH1* in SLEP revealed a weak association to BD (not shown). It is also worth noting that several PRCCGs not found to be illness-associated in SLEP have nevertheless been implicated in BD and/or related phenotypes by other studies. Examples include *MARCKS* [52], *BCL2* [53], *FKBP5* [54], and *CDKN1A* [55], which have been implicated in lithium's mechanism of action, apoptosis, glucocorticoid signaling, and neurogenesis respectively. In this context, it is tempting to speculate that the clock may connect several disparate biological processes thought to contribute to BD and the related spectrum illnesses.

While the clock modulators were not significantly over-represented among genes associated with psychiatric disorders, the genes highlighted by this work may nonetheless be worthy of future study. Of the ten clock modulator genes that are both illness-associated and lithium-responsive, most are expressed in the mouse brain (Allen Brain Atlas), and a majority of these are highly expressed in the hippocampus, a structure implicated in both mood disorders and lithium response. Also noteworthy is that *SH3GL2* and *FHIT*, two of the clock modulator genes that are both illness-associated and lithium-responsive, are located within two chromosomal areas (9p22.2 and 3p14) associated with comorbid BD and alcoholism [56]. Thus, it would be premature to dismiss upstream clock modulator genes as contributors to human psychopathology.

Taken together, our findings encourage the continued study of clock gene variants and their role in BD, lithium response, and related mental illnesses. However, our work also raises the problem of focusing too narrowly on individual genetic variants that, while functional, may be have relatively weak effects in isolation. Our work suggests that biological characterization of genetic variants of interest may be best accomplished using pathway based approaches that can assess overall function of a system, considering simultaneously multiple inputs that may impact upon it. In this respect, the circadian clock is well suited for further study, as molecular alterations in clock genes cause measurable changes in gene network function in single cells (e.g. gene expression rhythms), consequences for physiological process measured in tissues (e.g. endocrine rhythms), and consequences for behaviors that can be measured in whole animals or human patients (e.g. sleep/wake activity). Using this multi-level approach, genetic associations may reveal important nodes in the system that can be better characterized in functional studies.

## Supporting Information

Table S1 (XLS)

Table S2 (XLS)

Table S3 (XLS)
